# Distinct Requirements for Adaptor Proteins NCK1 and NCK2 in Mammary Gland Development

**DOI:** 10.1007/s10911-023-09541-1

**Published:** 2023-07-21

**Authors:** Adam P. Golding, Benjamin Ferrier, Laura A. New, Peihua Lu, Claire E. Martin, Erka Shata, Robert A. Jones, Roger A. Moorehead, Nina Jones

**Affiliations:** 1 Department of Molecular and Cellular Biology, University of Guelph, Guelph, ON Canada; 2 Present address: Lunenfeld-Tanenbaum Research Institute, Toronto, ON Canada; 3 Department of Biomedical Sciences, University of Guelph, Guelph, ON Canada

**Keywords:** Mammary gland development, Actin cytoskeleton, NCK, Branching morphogenesis, Knockout mice

## Abstract

The adaptor proteins NCK1 and NCK2 are well-established signalling nodes that regulate diverse biological processes including cell proliferation and actin dynamics in many tissue types. Here we have investigated the distribution and function of *Nck1* and *Nck2* in the developing mouse mammary gland. Using publicly available single-cell RNA sequencing data, we uncovered distinct expression profiles between the two paralogs. *Nck1* showed widespread expression in luminal, basal, stromal and endothelial cells, while *Nck2* was restricted to luminal and basal cells, with prominent enrichment in hormone-sensing luminal subtypes. Next, using mice with global knockout of *Nck1* or *Nck2*, we assessed mammary gland development during and after puberty (5, 8 and 12 weeks of age). Mice lacking *Nck1* or *Nck2* displayed significant defects in ductal outgrowth and branching at 5 weeks compared to controls, and the defects persisted in *Nck2* knockout mice at 8 weeks before normalizing at 12 weeks. These defects were accompanied by an increase in epithelial cell proliferation at 5 weeks and a decrease at 8 weeks in both *Nck1* and *Nck2* knockout mice. We also profiled expression of several key genes associated with mammary gland development at these timepoints and detected temporal changes in transcript levels of hormone receptors as well as effectors of cell proliferation and migration in *Nck1* and *Nck2* knockout mice, in line with the distinct phenotypes observed at 5 and 8 weeks. Together these studies reveal a requirement for NCK proteins in mammary gland morphogenesis, and suggest that deregulation of *Nck* expression could drive breast cancer progression and metastasis.

**Supplementary Information:**

The online version contains supplementary material available at 10.1007/s10911-023-09541-1.

## Introduction

Mammary development occurs through a process known as branching morphogenesis, where cells within the developing gland undergo extensive proliferation and migration to invade the surrounding fat pad and create the elaborate arborized network of the mature gland [1]. Epithelial cells compose the ducts and are surrounded by basement membrane, while the fat pad stroma is more diverse, containing fibroblasts, adipocytes, immune cells, blood vessels, and nervous system components in addition to the fibrous extracellular matrix (ECM) [1]. During puberty, estrogen triggers elongation of the rudimentary ductal tree, and stratified bulb-shaped structures known as terminal end buds (TEBs) form at the leading edge of the developing ducts. Each TEB contains a layer of cap cells at the tip, which differentiate into the outer basal layer of myoepithelial cells as the duct extends, in addition to several body cell layers, which differentiate into a single layer of luminal cells lining the ductal lumen [2]. Bifurcation of TEBs allows formation of new primary ducts which penetrate and fill the immature fat pad [1, 2].

Branching morphogenesis in the mammary gland is regulated by cell processes including collective migration and epithelial-to-mesenchymal transition (EMT), which are proposed to allow forward movement and invasion of epithelial cells into the fat pad during ductal elongation, along with expression of matrix metalloproteases, which promote degradation and path-clearing of the surrounding basement membrane and ECM [2]. In turn, the ECM and stroma contribute to mammary gland development by providing physical support, an adhesive substrate and a variety of signalling molecules. Reciprocal signalling between epithelial cells and the surrounding fat pad during mammary branching involves hormones, growth factors, cell surface receptors and adhesion molecules. In addition to hormone receptor signalling, activation of receptor tyrosine kinases (RTKs) such as the epidermal growth factor receptor (EGFR) enhances the proliferative and survival capacity of mammary epithelial cells [3, 4]. RTKs can also crosstalk with integrins, which couple the ECM to the actin cytoskeleton at focal adhesions. Integrin signalling controls cell adhesion, migration and invasion as well as EMT through activation of various effector proteins such as focal adhesion kinase (FAK) which converge on the actin cytoskeleton, and several studies have demonstrated the importance of this signalling axis in mammary development [5–7].

Here we investigate the role of Nck1 and Nck2 adaptor proteins in mammary gland branching morphogenesis. Nck (non-catalytic region of tyrosine kinase) proteins are broadly expressed and they regulate diverse biological processes including actin cytoskeleton organization and cell proliferation in many tissue types through their associations with both RTKs and integrins [8, 9]. Genetic studies have previously established that Nck proteins are essential for embryonic development and functionally redundant [10], though recent evidence suggests potentially divergent roles in vitro [11] and in vivo [12]. Furthermore, we and others have reported a role for Nck proteins in branching morphogenesis during development of the vascular system and in cardiac valve EMT [13–15]. In the present study, we profile expression of *Nck1* and *Nck2* in the mouse mammary gland, and using knockout mice, we demonstrate distinct temporal requirements for Nck proteins in ductal branching, TEB formation and cell proliferation.

## Materials and Methods

### Mouse Strains 

Mice with systemic *Nck1* knockout (B6.Cg-Nck1 < tm1 > Paw) or *Nck2* knockout (B6.Cg-Nck2 < tm1 > Paw) [10] were bred for more than 10 generations onto the C57BL/6 J background (kindly provided by Dr. Tony Pawson, Toronto, ON, Canada). *Nck1* and *Nck2* single knockout mice on this background are viable and have been described previously [12]. Mice were housed in a 14-h light/10-h dark cycle with free access to food and water. Genotyping was performed by PCR using primer sequences listed in Supplemental Table 1 [10]. Only virgin female mice were used in this study. Mice were euthanized by CO_2_ inhalation. All animal studies were approved by the Animal Care Committee at the University of Guelph (AUP #4174) and complied with the Canadian Council of Animal Care guidelines.

### Mammary Gland Processing and Wholemount Staining

At indicated ages, tissue extraction was performed where number four inguinal mammary glands were removed and placed on glass slides to be air-dried for 5–10 min. Slides were immediately fixed in Clarke’s Solution (75% absolute ethanol and 25% glacial acetic acid). The next day, slides were removed from Clarke’s Solution and dehydrated in 70% ethanol for 30 min. Slides were then placed in carmine alum solution (0.5 g carmine with 1.25 g aluminum potassium sulphate dissolved in 120 mL of boiling H_2_O while stirring in fume hood) overnight. Slides were destained if necessary in destain solution (97% absolute ethanol and 3% HCl). The following day, slides were dehydrated in increasing ethanol concentrations (70%, 90%, 100%) for 15 min each. Slides were then placed in xylene to remove fat from the mammary gland and kept in xylene until imaged, after which point the gland could be dried. Images were captured with a Canon EOS 6D camera equipped with a 100 mm Canon macro lens (Canon Canada, Mississauga, ON).

### Wholemount Mammary Gland Analysis

All analysis of mammary gland wholemounts was performed in FIJI (Fiji is just ImageJ) from images taken at 1 × magnification. Quantification of length from the back of the lymph node to the 3–4 furthest extensions of the ductal network represents mammary gland outgrowth (mm). Quantification of ductal branch area (mm^2^) represents the total ductal area extending from a perpendicular line drawn at the back of the lymph node. Overall branch point frequency was determined after counting the total length of the ductal system in a representative area, and the number of branch points therein. TEBs were quantified as any bulbous structure at the leading edge of the ductal branch network with a width > 100 μm to ensure consistency between samples. TEBs undergoing bifurcation were excluded from the analysis. Data were analysed via FIJI and Excel.

### Immunohistochemistry

The fourth or matching opposite ninth mammary gland was extracted from mice at 5, 8 and 12 weeks of age and placed into a cassette incubated overnight in 10% formalin. After 24 h, the cassette was moved to 70% ethanol and then embedded into paraffin by the University of Guelph Animal Health Laboratory. Sections at 4 μm thickness were prepared from the paraffin block, fixed to charged glass slides and allowed to dry overnight in a 37 °C oven. The following day, slides were washed 3 × 5 min with xylene to deparaffinize the tissue and then hydrated by washing 3 × 2 min in 100% isopropanol, 1 × 2 min in 95% isopropanol, 1 × 2 min in 70% isopropanol, and finally 1 × 2 min in deionized water. Antigen retrieval was next performed using Dako Target Retrieval solution, pH 6 (Agilent) for 15 min at 95 °C in a Decloaking Chamber (Biocare). After washing in deionized water, slides were placed in 3% hydrogen peroxide for 10 min at room temperature to block endogenous peroxidase activity. Immunohistochemistry was performed at room temperature using Dako Autostainer Link 48 instrument (Agilent). Briefly, sections were blocked in Dako universal serum-free protein block (Agilent) for 12 min, then incubated with rabbit anti-Ki67 antibody (New England Biolabs D3B5, Mouse preferred/ IHC formulated) at 1:400 concentration for 30 min, followed by Dako EnVision + anti-rabbit HRP-labelled polymer (Agilent) for 30 min. Vector Nova Red chromogen (Vector Laboratories) was used as HRP substrate for 10 min, followed by hematoxylin counterstain for 3 min.

### Ki67 Analysis

Ki67 positivity was quantified through analysis of images taken with a Leica DM1000 microscope and an OMAX A3580U camera with ToupView program at 40 × magnification using Extended Depth of Field (EDF). TIFF quality images were transferred into QuPath program (Version 1.2) available at https://qupath.github.io/. Cell detection in Qupath program was performed using the default settings and then detected cells were processed through a “classifier” based on the “Dab test script” available online. Cell counts and measurements were exported through detection measurements function, saved and data was compiled into Excel. Final Ki67 positivity per TEB was calculated as the percentage of DAB positive cells. For each animal, a minimum of 5 TEBs (range 5–11) were analyzed.

### RNA Isolation and Quantitative PCR

Total RNA was isolated from the third or matching opposite eighth mammary glands (representative region adjacent to the lymph node) of 5, 8 and 12-week old female mice using the Purelink™ RNA Mini Kit (Invitrogen™; Catalog #12183018A) and treated with DNase 1 (Invitrogen). Reverse transcription was performed with 1 μg extracted RNA using qScript™ cDNA SuperMix (QuantaBio; Catalog #CA 101414–104). qRT-PCR reactions were carried out using PerfeCTa® SYBR green FastMix® with ROX™ (Quanta Biosciences; Catalog #95,073–250) on a StepOnePlus™ Real-Time PCR system (Applied Biosystems). Expression differences between samples were calculated using the delta-Ct method. Differences were normalized to reference genes *Hprt* and *Gapdh*. For droplet digital PCR, sample cDNA was first diluted to give an optimal droplet count number for each specific gene. cDNA was mixed with 2.75 μmol of each primer and QX200 ddPCR EvaGreen Supermix (1,864,033, BioRad). Droplets were generated using Automated Droplet Generation Oil for EvaGreen and QX200 AutoDG ddPCR System (BioRad). Droplets were read using QX200 Droplet Reader and analysed using QuantaSoft software (BioRad). Values were normalized to *Hprt* and *Gapdh* levels. Primer sequences utilized for the analysis are listed in Supplemental Table 2 and were designed in-house or selected from PrimerBank [16].

### Online Gene Expression Databases

Mouse mammary gland RNA sequencing data for *Nck1* and *Nck2* were compiled from the web tool of the publicly available *Tabula Muris* single cell transcriptome project (https://tabula-muris.ds.czbiohub.org) [17] or retrieved from the Marioni Lab database using their web tool (https://marionilab.cruk.cam.ac.uk/mammaryGland/) [18].

### Statistics

Comparisons of three or more groups were done using one-way ANOVA with post-hoc Dunnett’s multiple comparison test where star designation indicated significance (* *p* < 0.05; ** *p* < 0.01; *** *p* < 0.001; **** *p* < 0.0001). Comparisons between two groups were performed using a Mann–Whitney test where star designation indicated significance (* *p* < 0.05; ** *p* < 0.01). Statistical analysis and graph preparations were performed using GraphPad Prism (v8 or v9). Data are presented as mean ± standard error.

## Results

### *Nck1 *and *Nck2* are Expressed in Various Cell Types in the Developing Mouse Mammary Gland

**Fig. 1:**
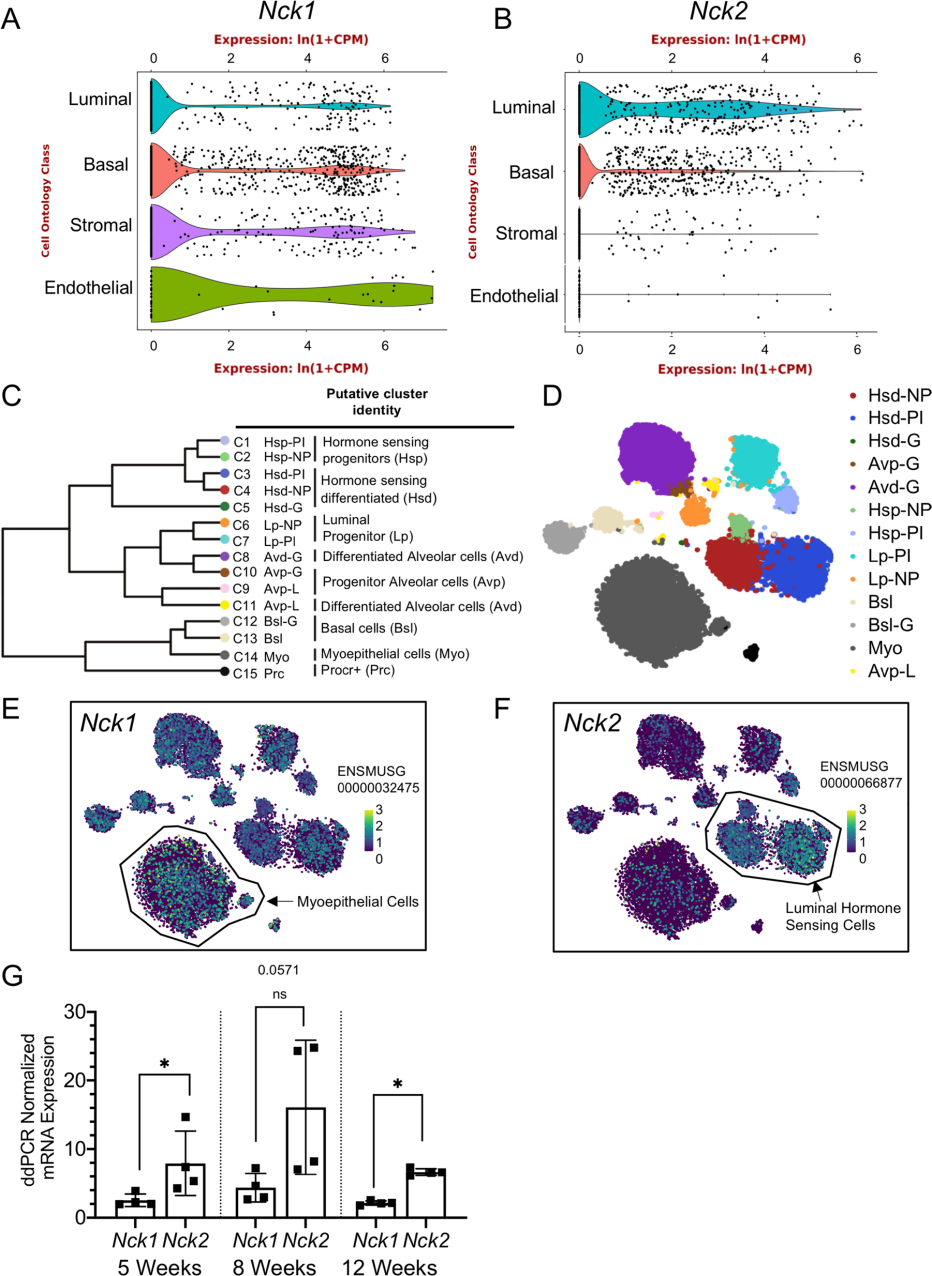
*Nck1* and *Nck2* are expressed in various cell types in the developing mammary gland. **A**, **B** Single cell RNA sequencing of *Nck1* and *Nck2* in all cell types of the murine mammary gland (from Schaum et al. 2018) [17]. **A** Violin plot of *Nck1* gene expression in clustered groups defined as luminal epithelial cells, basal cells, stromal cells, and endothelial cells of the mammary gland. **B** Violin plot of *Nck2* gene expression in clustered groups defined in (**A**). **C**, **D** (**C**) Dendrogram showing putative identities of 15 distinct mammary epithelial cell clusters following single cell RNA sequencing of nulliparous “NP”, mid gestation “G”, lactation “L”, and post-involution “PI” mammary glands and (**D**) t-SNE plot of mammary epithelial cell clusters used to overlay with *Nck1* (**E**) and *Nck2* (**F**) expression (from Bach et al. 2017) [18]. **E** *Nck1* gene expression was detectable in all clusters, with highest levels in the myoepithelial C14 cluster. **F** High levels of *Nck2* gene expression were detectable primarily in luminal hormone sensing progenitors C1 and C2 and hormone sensing differentiated C3 and C4 clusters. **G** ddPCR analysis of *Nck1* and *Nck2* abundance, normalized to *Hprt/Gapdh*, in 5, 8 and 12 week old wildtype mouse mammary glands of the indicated genotype (*n* = 4). * *P* < 0.05; by Mann–Whitney test performed independently at each of the respective timepoints (5, 8, and 12 weeks)

To begin our analysis of *Nck1* and *Nck2* function in mammary gland development, we first profiled their expression using publicly available online resources. Analysis of single cell transcriptome data from 8-week old mouse mammary gland tissue in the *Tabula Muris* compendium [17] revealed widespread expression of *Nck1* in luminal, basal, stromal and endothelial cells, while *Nck2* transcripts were restricted to luminal and basal cells (Fig. 1A, B). Similarly, analysis of subgroups of luminal and basal mammary epithelial cells identified by single cell RNA sequencing of mouse mammary glands by the Marioni and Khaled laboratories [18] confirmed the distinct profiles for *Nck1* and *Nck2* (Fig. 1C-F). *Nck1* expression was readily detected in all subgroups, with highest levels seen in myoepithelial cells (Fig. 1E). By contrast, *Nck2* was predominantly expressed in terminally differentiated hormone-sensing luminal cells which possess high levels of hormone receptors and showed lower expression in myoepithelial cells compared to *Nck1* (Fig. 1E, F). We also performed droplet digital PCR (ddPCR) to compare mRNA concentrations in the mammary gland at several key timepoints throughout mouse development. We found that *Nck2* was more abundant than *Nck1* at all timepoints, and that relative levels of each paralog increased from 5 to 8 weeks and then decreased at 12 weeks (Fig. 1G). Altogether our results show that *Nck1* and *Nck2* are broadly yet differentially expressed throughout the developing mammary gland.

### Loss of *Nck1 *or *Nck2* Delays Ductal Outgrowth and Reduces Total Branch Area

**Fig. 2:**
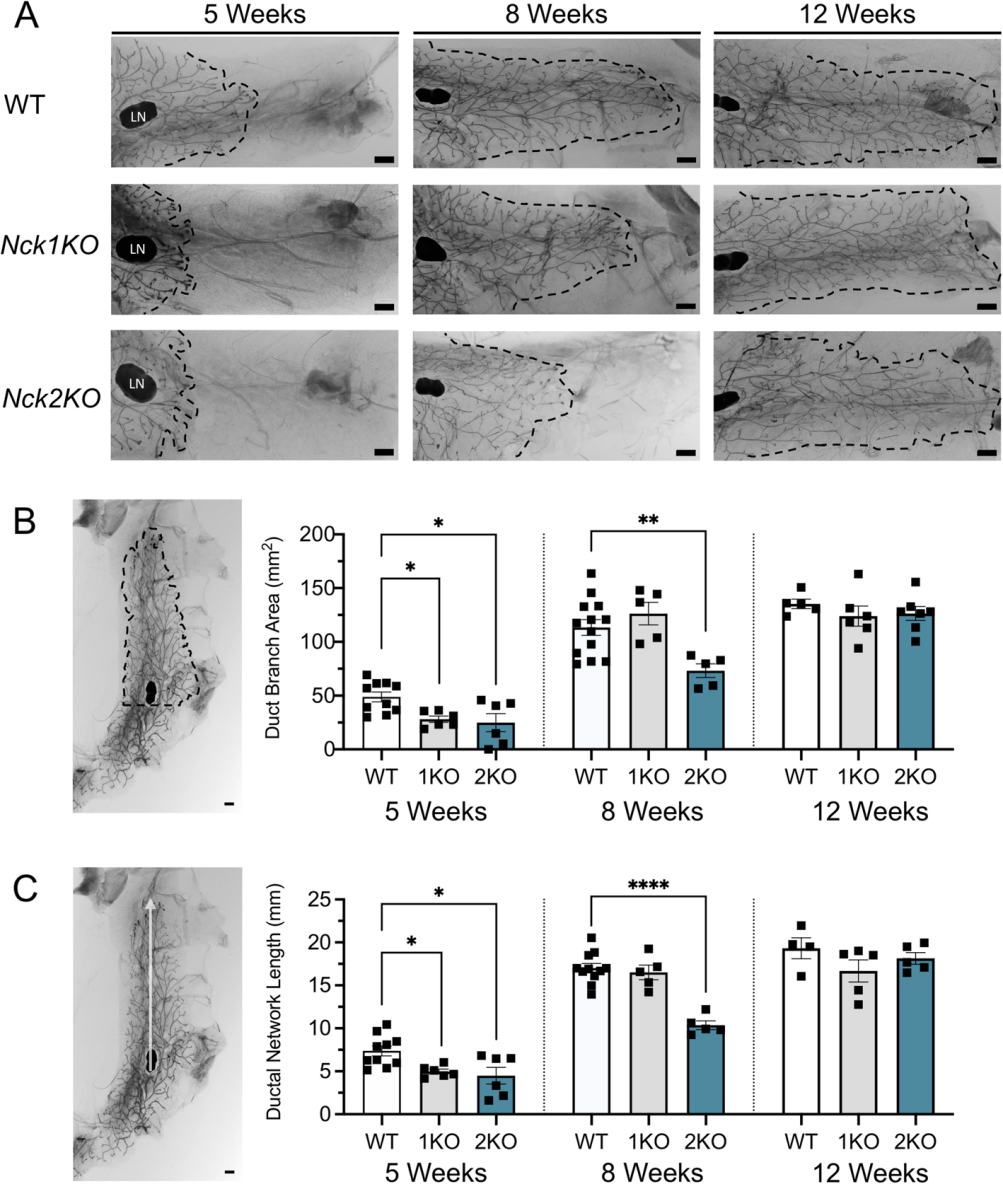
Loss of *Nck1* or *Nck2* delays ductal outgrowth and total branch area at 5 and 8 weeks. **A** Representative wholemount images of L4 mammary gland outgrowth in WT, *Nck1KO*, and *Nck2KO* mice at 5 weeks, 8 weeks, and 12 weeks of age. Scale bar 1 mm. **B** Ductal branch area was quantified using FIJI where a perpendicular line was drawn at the back of the lymph node (LN) and the full branching region extending from here was outlined (*n* = 5–10 per genotype). **C** Ductal outgrowth was quantified by averages measuring from the back of the lymph node (LN) to the 3–4 furthest extensions of the ductal network (*n* = 5–10 mice of each genotype per timepoint). B,C, 1KO: *Nck1KO*, 2KO: *Nck2KO*, scale bar 1 mm; * adjusted *P* < 0.05; ** *P* < 0.01; **** *P* < 0.0001; at each time point by one-way ANOVA with post-hoc Dunnett’s test of *Nck1KO* and *Nck2KO* compared to WT

Given the robust expression of *Nck1* and *Nck2* throughout the mammary gland, we next examined the effects of their deletion on mammary gland development using mice with total body knockout (KO) of *Nck1* or *Nck2* (*Nck1KO*/1KO or *Nck2KO*/2KO, respectively) [10] in comparison to wildtype (WT) controls. To assess the progressive invasion of the fat pad and ductal branching that occurs during mammary gland development, we performed wholemount analyses on L4 mammary glands isolated from 5, 8 and 12 week old virgin female mice of each genotype (Fig. 2A). These timepoints represent established milestones for mammary gland development in C57BL/6 mice [19, 20] and there were no differences in body weight between genotypes at each timepoint (Supplemental Fig. 1). At 5 weeks of age (early puberty), there was significantly less ductal outgrowth and ductal branch area in both *Nck1KO* and *Nck2KO* mice relative to WT (Fig. 2B, C). At 8 weeks of age (late puberty), the defects in invasion and branching persisted in *Nck2KO* mice compared to WT, while there were no significant differences between *Nck1KO* and WT mice (Fig. 2B, C). In mature 12 week old animals, there were no significant differences in ductal outgrowth or branch area among WT, *Nck1KO*, and *Nck2KO* mice (Fig. 2B,C). These latter findings are consistent with the ability of *Nck1KO* and *Nck2KO* mothers to lactate and support the growth of their pups (data not shown). Collectively these findings indicate that Nck1 and Nck2 are required during early mammary gland development, and that compensation occurs to allow recovery of fully functional glands in adult mice.

### Loss of *Nck1 *or *Nck2* Differentially Impact TEB Number During Development

**Fig. 3:**
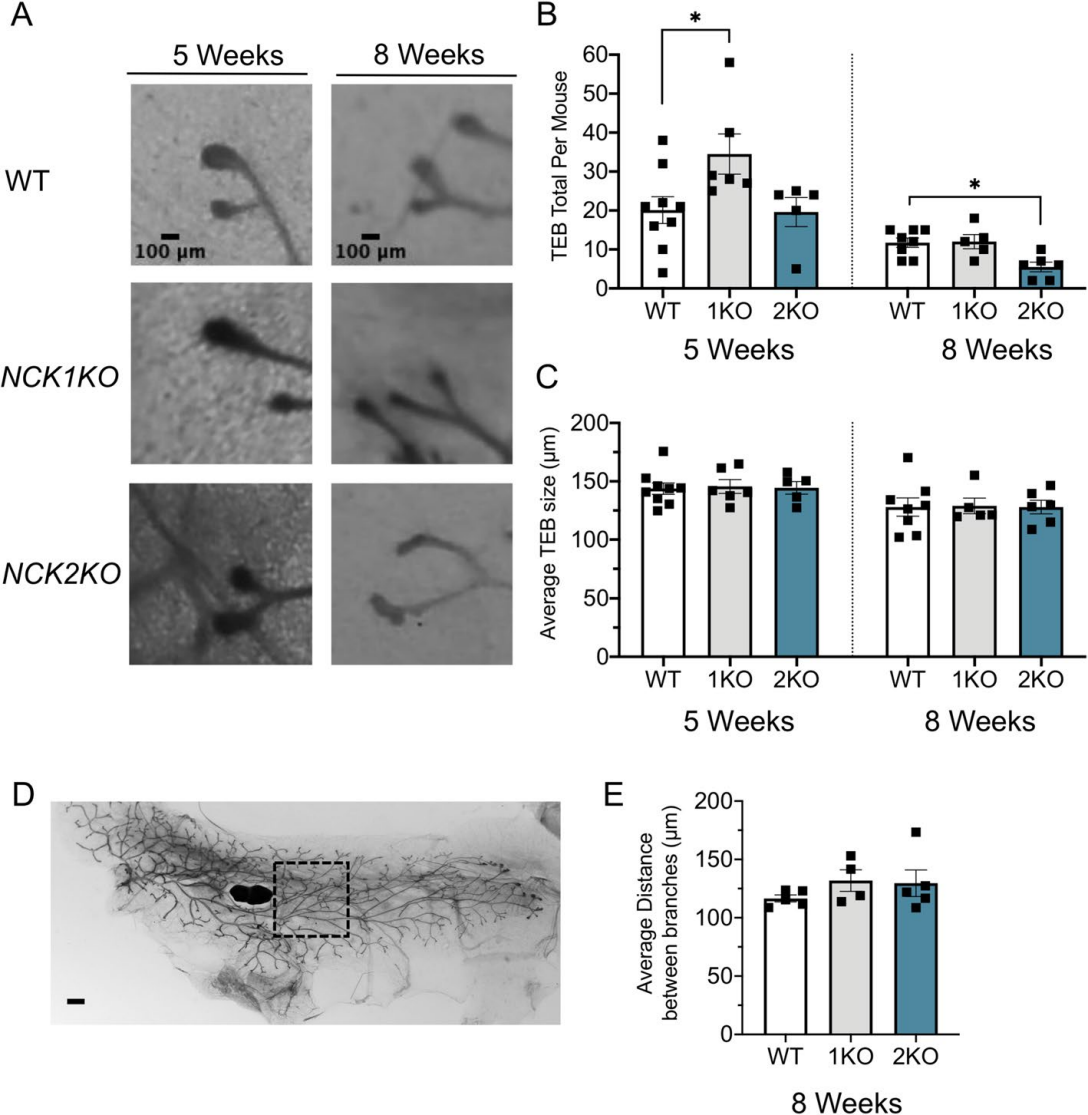
*Nck1* and *Nck2* have opposing effects on TEB number at 5 and 8 weeks of age, with no effect on TEB size. **A** Representative high magnification images of TEBs acquired from mammary gland wholemounts prepared from WT, *Nck1KO* and *Nck2KO* mice at 5 weeks (left) and 8 weeks (right). Scale bar 100 μm. **B** Quantification of total TEB number per mouse at 5-week and 8-week timepoints. TEB minimum size was set at 100 μm to ensure consistency across samples (*n* = 5–10 mice of each genotype). TEBs undergoing bifurcation were excluded from the analysis. **C** Quantification of average TEB size per mouse in each genotype at 5-week and 8-week timepoints. **D** Representative image showing area selected for quantitation of branching rate. Scale bar 1 mm. **E** Quantification of branching rate (average distance between branches) in 8-week old mice (*n* = 4–5 mice of each genotype), counted as the total length of the ductal system divided by the number of branch points in the area measured in (**D**). B,C,E, 1KO: *Nck1KO*, 2KO: *Nck2KO*, * adjusted *P* < 0.05; at each time point by one-way ANOVA with post-hoc Dunnett’s test of *Nck1KO* and *Nck2KO* compared to WT

Mammary gland branching and outgrowth processes are driven in part by TEBs, which appear at the leading edge during puberty and undergo repeated rounds of bifurcation to form the arborized ductal network [2]. To quantitate TEB number, size and branch point frequency, we performed morphometric analysis on mammary gland wholemounts prepared from mice at 5 and 8 weeks of age from each genotype (Fig. 3A). At the 5-week timepoint, we observed an increase in TEB number in *Nck1KO* mice but not *Nck2KO* mice compared to WT (Fig. 3B), yet there was no difference at 8 weeks for *Nck1KO* mice compared to WT mice (Fig. 3B). Conversely, in *Nck2KO* mice, there was a significant reduction in TEB number at 8 weeks compared to WT mice (Fig. 3B). At both timepoints, there were no significant differences in TEB size across the three genotypes (Fig. 3C). Similarly, there were no differences in the rate of branching among the genotypes at 8 weeks of age (Fig. 3D, E). These results highlight distinct roles for Nck1 and Nck2 in TEB formation during mammary morphogenesis.

### Loss of *Nck1 *or *Nck2* Disrupts Mammary Epithelial Cell Proliferation

**Fig. 4:**
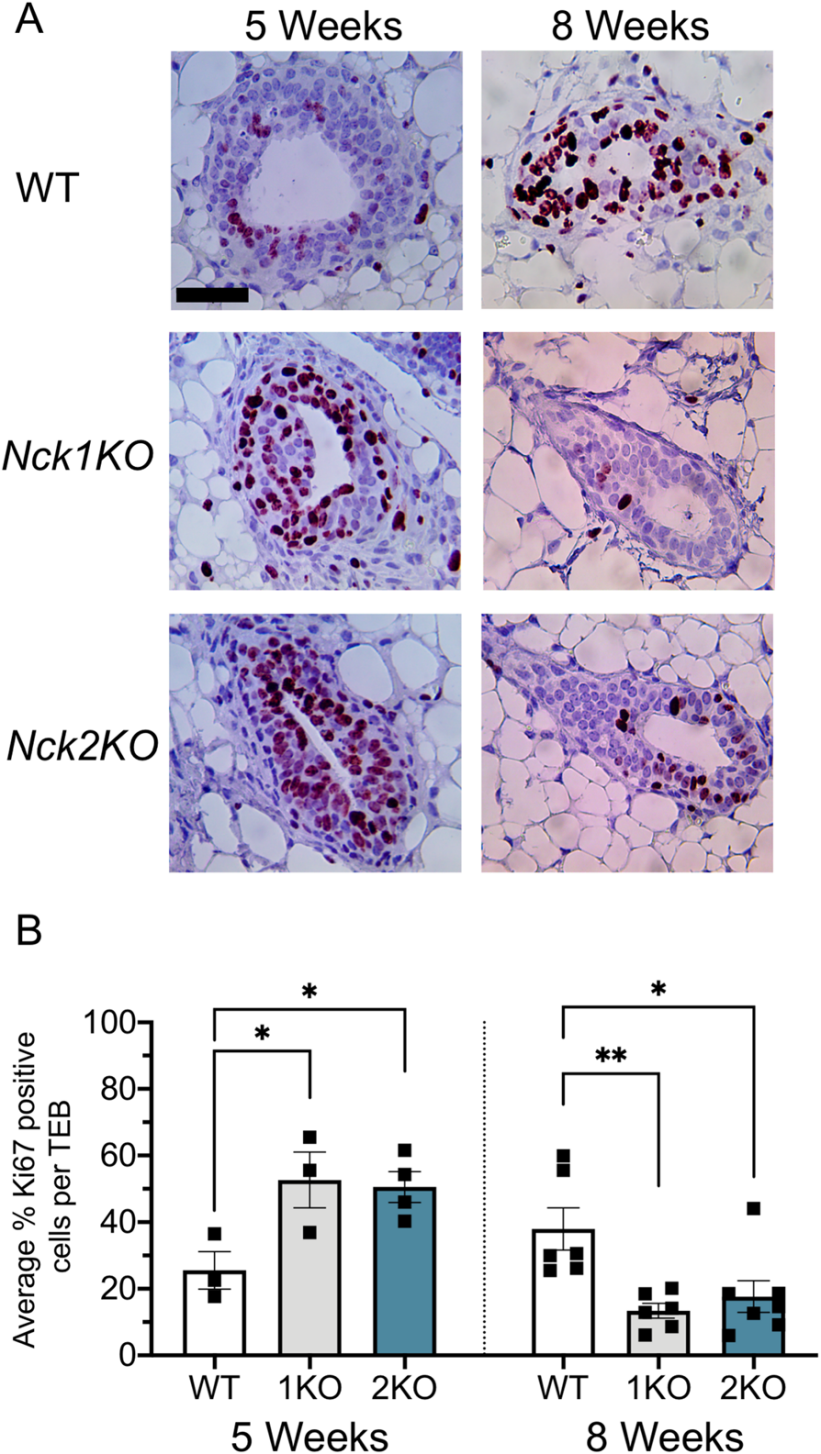
Loss of *Nck1* or *Nck2* disrupts mammary epithelial cell proliferation. **A** Representative images of TEB Ki67 immunostaining on mammary gland sections prepared from 5 and 8 week old WT, *Nck1KO*, and *Nck2KO* mice. Scale bar 50 μm. **B** Quantification of average Ki67 positive cell percentage per TEB (*n* = 5–11 TEBs per mouse) at 5 weeks (*n* = 3–4 mice per genotype) and 8 weeks (*n* = 6–7 mice per genotype). 1KO: *Nck1KO*, 2KO: *Nck2KO*, * adjusted *P* < 0.05; ** *P* < 0.01; at each time point by one-way ANOVA with post-hoc Dunnett’s test of *Nck1KO* and *Nck2KO* compared to WT

Given the defects in ductal morphogenesis observed in *Nck1KO* and *Nck2KO* mice during outgrowth, we next investigated the proliferation status of the mammary epithelium. We performed immunohistochemical staining of mammary gland sections at 5 and 8 weeks of age using Ki67 as a nuclear marker of cell proliferation and assessed proliferation levels within TEBs (Fig. 4A). Quantitation of Ki67-positive nuclear area revealed a significant increase in the percentage of Ki67-positive cell area in TEBs of both *Nck1KO* and *Nck2KO* mice at 5 weeks compared to WT controls, with a concomitant significant decrease at 8 weeks in both *Nck1KO* and *Nck2KO* mice (Fig. 4B). Together these results suggest that Nck1 and Nck2 regulate proliferation of the mammary epithelium.

### Loss of *Nck1 *or *Nck2* Alters Gene Expression Patterns in the Developing Mammary Gland

**Fig. 5:**
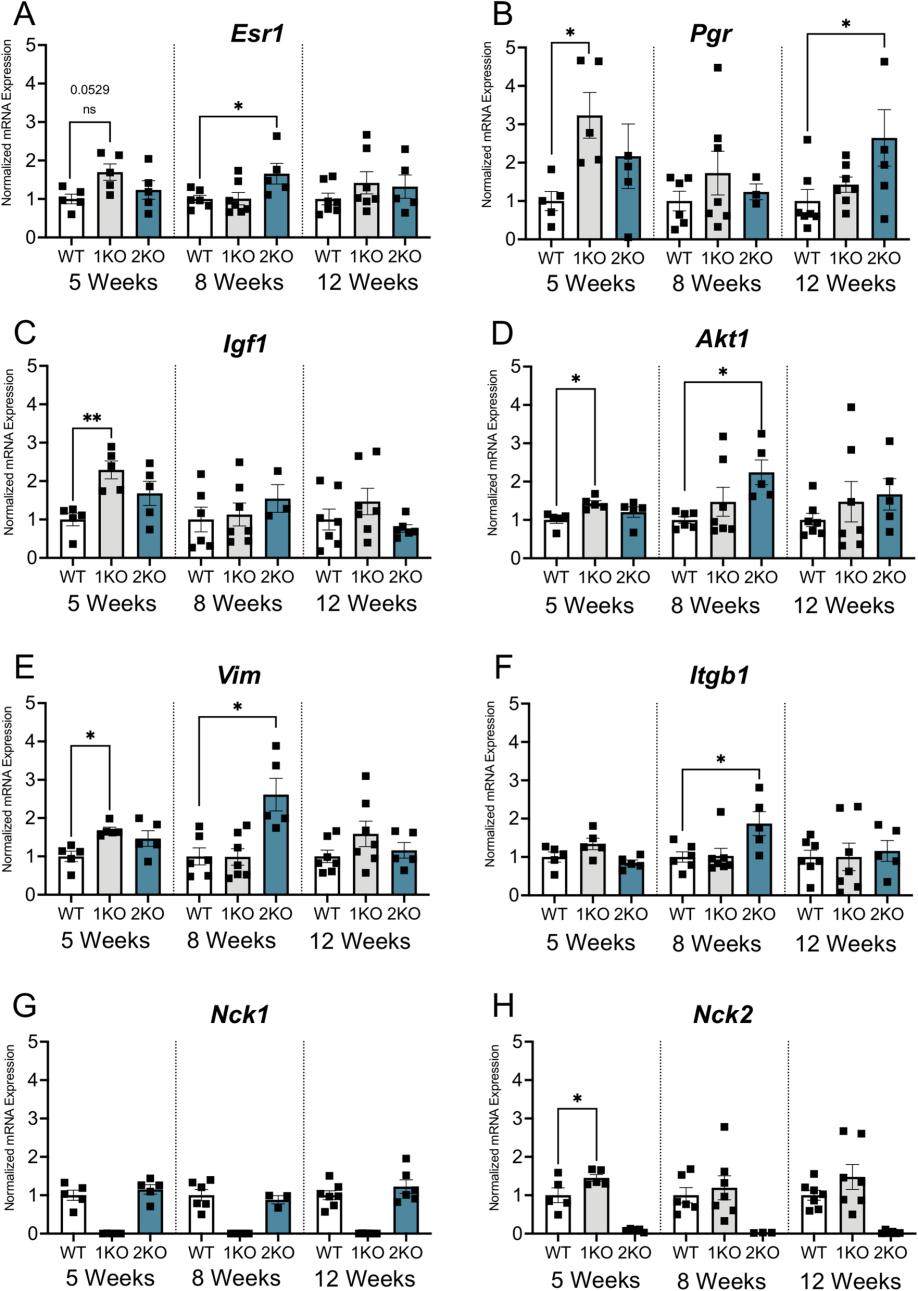
Loss of *Nck1* or *Nck2* alters gene expression patterns in the developing mammary gland. Quantitative PCR analysis of L3 mammary glands isolated from 5, 8 and 12 week old WT, *Nck1KO* and *Nck2KO* mice (*n* = 5–7 mice of each genotype). mRNA fold changes are shown for genes encoding (**A**) Estrogen Receptor α (*Esr1*), **B** Progesterone receptor (*Psr1*), **C** Insulin-like growth factor 1 (*Igf1*), **D** Akt1 (*Akt1*), **E** Vimentin (*Vim*), **F** Integrin β1 (*Itgb1*), **G** Nck1 (*Nck1*) and (**H**) Nck2 (*Nck2*). A-H, 1KO: *Nck1KO*, 2KO: *Nck2KO*, * adjusted *P* < 0.05, ** *P* < 0.01; at each time point by one-way ANOVA with post-hoc Dunnett’s test of *Nck1KO* and *Nck2KO* compared to WT

To characterize potential molecular mechanisms underlying the defects in mammary morphogenesis in *Nck1* and *Nck2* deficient mice, we analyzed expression of several key genes that have been previously determined to function in mammary gland development at our established 5, 8 and 12 week timepoints. As proliferation in the mammary gland is driven by hormone-dependent signalling, we first assessed levels of receptors for estrogen (*Esr1*) and progesterone (*Pgr*). We found that *Pgr1* was significantly upregulated at 5 weeks of age in *Nck1KO* mice, and a similar trend nearing significance was also seen with *Esr1* (Fig. 5A, B). Interestingly, this pattern of upregulation was also seen in *Nck2KO* mice, but not until later timepoints (Fig. 5A,B). We next surveyed expression of genes associated with mammary epithelial cell proliferation, migration and EMT. As we saw with *Esr1* and *Pgr1*, *Nck1KO* mice showed significant upregulation of *Igf1*, *Akt1* and *Vim* at 5 weeks of age, while *Nck2KO* mice showed this pattern at 8 weeks of age for *Akt1* and *Vim* (Fig. 5C-E). Notably, *Nck2KO* mice but not *Nck1KO* mice also showed robust significant increases in transcript levels of Integrin β1 (*Itgb1*) expression at the 8-week timepoint (Fig. 5F). Lastly, to investigate potential compensation between the paralogs, levels of *Nck1* and *Nck2* were assessed. We found that *Nck1* expression remained unchanged in *Nck2KO* mice compared to WT controls, while *Nck2* was significantly upregulated in *Nck1KO* mice at 5 weeks of age (Fig. 5G,H). Taken together, our results uncover changes in mammary gland gene expression in *Nck1KO* and *Nck2KO* mice at key stages of development, and they suggest that compensation from Nck2 in *Nck1KO* mice may support earlier resolution of the defects in *Nck1KO* mice.

## Discussion

**Fig. 6:**
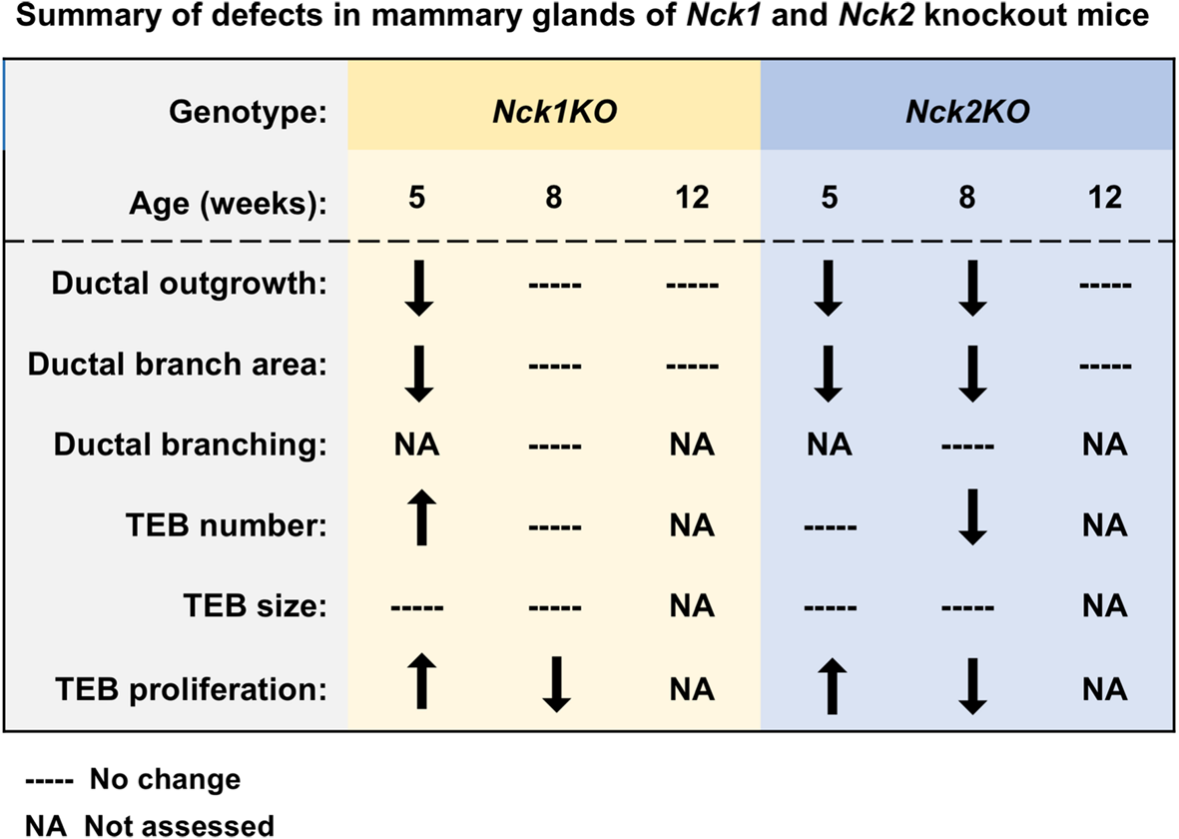
Summary of mammary gland phenotypes in *Nck1* and *Nck2* knockout mice at key stages of development. Mice lacking *Nck1* or *Nck2* show distinct temporal defects in ductal outgrowth, overall branch area, TEB number and TEB proliferation rates during puberty at 5 weeks and 8 weeks, and the outgrowth and branching phenotypes resolve by 12 weeks

In this report, we have investigated the distribution and function of *Nck1* and *Nck2* in the developing mammary gland. We found that ductal outgrowth and branch area were significantly impaired in both *Nck1KO* and *Nck2KO* animals in early puberty (5 weeks), and these defects persisted through to late puberty (8 weeks) in *Nck2KO* mice before normalizing at maturity (12 weeks) (summarized in Fig. 6). The profound negative effect of Nck deletion on mammary outgrowth and branching is not surprising given previous studies defining NCK1 and NCK2 as key modulators of actin dynamics [8, 21, 22]. These defects were attributed in part to altered signalling through the small GTPases CDC42, RAC1, and RHOA, which along with their associated regulatory proteins, have all been implicated in aspects of mammary gland development in both in vitro and in vivo settings [23–27]. Interestingly, we found changes in expression of several genes associated with migration and invasion, including *Itgb1*, *Akt1 and Vim* which were collectively increased in *Nck2KO* mice but not *Nck1KO* mice at 8 weeks of age. At this later timepoint, delays in outgrowth and branching were uniquely present in *Nck2KO* mice, suggesting that there may be a distinct requirement for NCK2 in branching morphogenesis in the mammary gland.

The defects in ductal morphogenesis seen in *Nck1KO* and *Nck2KO* mice may also be due to alterations in TEBs, wherein both migration and proliferation are required to drive their extension into the fat pad. In early puberty (5 weeks), *Nck1KO* mice but not *Nck2KO* mice produced more TEBs, and this corresponded with a striking increase in proliferation and expression of *Pgr, Esr1* and *Igf1*, which are all potent stimulators of TEB formation and mammary epithelial cell proliferation [28]. However, no changes in TEB size were noted in either genotype. This suggests that a counter effect of the increased proliferation stress in *Nck2KO* mice could be apoptosis, though our attempts to assess this were unsuccessful. In later puberty (8 weeks), *Nck2KO* mice but not *Nck1KO* mice had fewer TEBs, when both genotypes showed decreased epithelial cell proliferation. Although we cannot rule out the possibility that the drop in proliferation could be due to depletion of a progenitor cell population [29], since the overall frequency of branches being formed through bifurcation of TEBs was also unaltered, it seems more likely that the persistent delay in outgrowth in *Nck2KO* mice at this timepoint results in fewer potential locations for TEBs to form.

The phenotypes of *Nck1KO* and *Nck2KO* mice differed somewhat, which is likely reflective of their distinct expression profiles in the developing mammary gland. During puberty, *Nck1* was broadly detected in epithelial and stromal cells, while *Nck2* was restricted to epithelial cells, particularly the hormone-sensing luminal subgroup. Total *Nck2* levels were also consistently higher than *Nck1* at all timepoints. Interestingly, expression of *Nck2* was increased in *Nck1KO* mice, most noticeably at 5 weeks of age, and this compensatory response may explain the earlier recovery of outgrowth and branching defects in mice lacking Nck1. In *Nck2KO* mice, the absence of Nck2 in cells that respond to hormones during puberty may underlie the persistent branching defect at 8 weeks of age, though compensation through responses such as increased *Pgr* expression may allow the ductal network to catch up by 12 weeks of age. Future studies using transplantation and/or conditional deletion of *Nck1* and *Nck2* will be required to provide further insight into lineage-specific and paralog-specific roles of these adaptor proteins. It should also be considered that the germline gene deletions could indirectly affect mammary gland development in the knockout mice, for instance by disrupting cell function in stromal cell populations and/or adipose tissue, or by altering production of ovarian hormones that influence sexual maturation. Indeed, we acknowledge the possibility that aspects of the observed phenotypes in *Nck1KO* and *Nck2KO* mice could be the result of a delay in the onset of puberty and therefore delayed puberty-induced ductal elongation. Nonetheless, our findings suggest that the NCK paralogs direct non-redundant signalling pathways during mammary gland development.

Similar defects in ductal outgrowth have been reported for NCK-associated signalling proteins, including EGFR [30], SRC [31], FAK [32], and Integrin β1 [33]. Interestingly, we detected changes in *Itgb1* expression in *Nck2KO* mice but not *Nck1KO* mice at 8 weeks of age, coincident with the persistent branching defect. Previous studies have shown that selective deletion of Integrin β1 from basal cells results in disorganized ductal branching and decreased side branching [33], and that application of an Integrin β1 directed antibody impairs ductal outgrowth [34]. Furthermore, perturbation of Integrin β1 function in the mammary epithelium leads to growth defects associated with altered SHC and AKT phosphorylation [35]. In line with this, changes in *Itgb1* expression in *Nck2KO* mice correlated with altered *Akt1* levels. While it remains to be determined whether these changes reflect an attempt to restore corresponding deficiencies in protein expression, it is tempting to speculate that NCK2 could mediate the cross-talk between adhesion signalling and growth factor activity that occurs during normal gland development. Indeed, the unique ability of Nck2 to associate with the integrin-linked kinase (ILK)-PINCH-PARVIN complex and thereby bridge the tail of Integrin β1 with activated EGFR [36] provides further support to explain the protracted requirement for NCK2 in branching morphogenesis.

In summary, NCK1 and NCK2 functions have been characterized in several in vivo settings associated with branching, including embryogenesis [10], angiogenesis [10, 13], and podocyte foot process formation and maintenance [12, 37, 38], and the studies herein now reveal a role for NCK1 and NCK2 in mammary duct morphogenesis. Analysis of the molecular mechanisms which control mammary gland development is essential, as these signalling pathways involving hormone-mediated proliferation and actin cytoskeleton remodeling can become deregulated and serve as promoters of breast cancer [7, 39, 40]. Notably, during the course of this investigation, NCK1 and NCK2 were identified as drivers of breast cancer progression and metastasis [41, 42]. Further investigation is needed to understand the mechanisms by which NCK1 and NCK2 signalling in development may be abnormally activated in breast cancer.

## Supplementary Information

**Additional file 1:** **Figure 1.** Body weights of WT, *Nck1KO*, and *Nck2KO* B6 mice at timepoints 5, 8, and 12 weeks. **Table 1.** Mouse genotyping primers. **Table 2.** Primer information for quantitative PCR.
