# Supplementary material for: Distinct Requirements for Adaptor Proteins NCK1 and NCK2 in Mammary Gland Development
Source: J Mammary Gland Biol Neoplasia. 2023 Jul 21;28(1):19. doi: 10.1007/s10911-023-09541-1 (PMC10361900; doi:10.1007/s10911-023-09541-1)
Supplement: Supplementary file 1 — Additional file 1: Figure 1. Body weights of WT, Nck1KO, and Nck2KO B6 mice at timepoints 5, 8, and 12 weeks. Table 1. Mouse genotyping primers. Table 2. Primer information for quantitative PCR. [file 10911_2023_9541_MOESM1_ESM.pdf]

Distinct Requirements for Adaptor Proteins Nck1 and Nck2 in Mammary Gland Development.

Adam Golding et al. 2023. Journal of Mammary Gland Biology and Neoplasia.

DOI: 10.1007/s10911-023-09541-1

Supplemental Materials Table of Contents

Supplemental Figure 1: Body weights of WT, *Nck1KO*, and *Nck2KO* B6 mice at timepoints 5, 8, and 12 weeks.

Supplemental Table 1: Mouse genotyping primers

Supplemental Table 2: Primer information for quantitative PCR

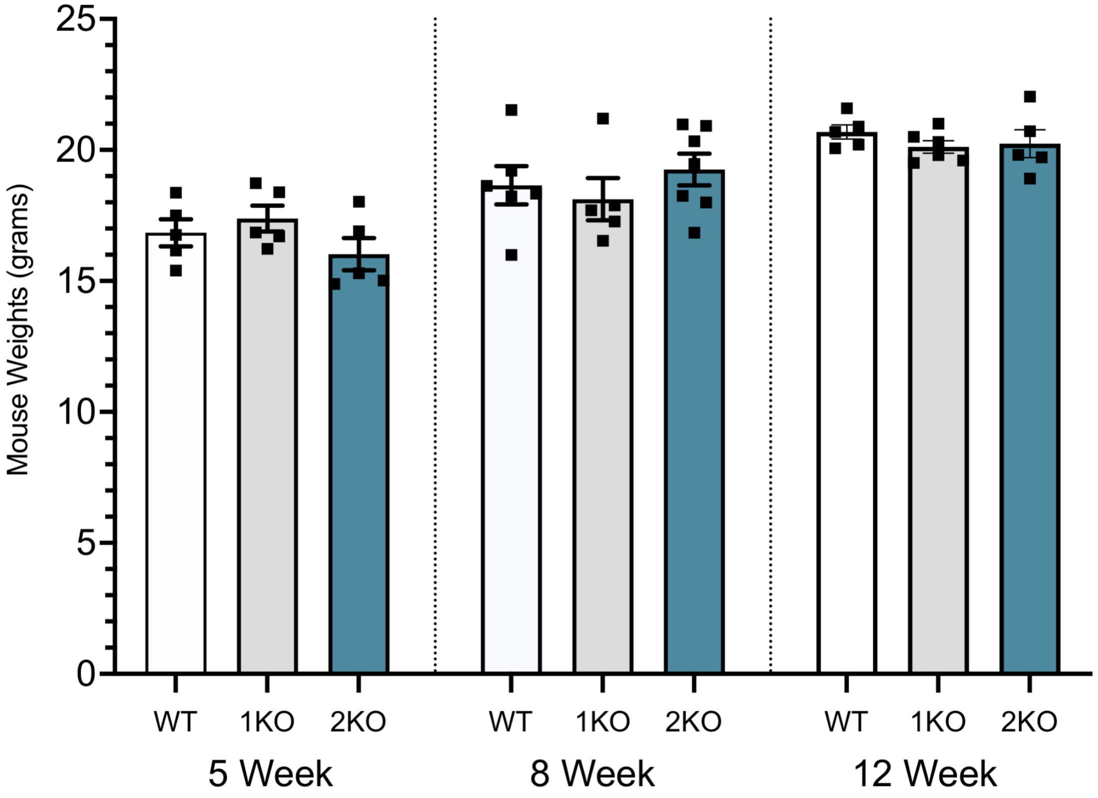

Supplemental Figure 1. Body weights of WT, *Nck1KO* (1KO), and *Nck2KO* (2KO) B6 mice at timepoints 5, 8, and 12 weeks respectively (n=5-7 per genotype). Mouse weights were measured at endpoint in grams.

Supplemental Table 1: Mouse genotyping primers

Nck1 WT Fw	gcatgtagacaattacacttcagcacc
Nck1 WT Rv	attcatggaatttcgaactcgccacc
Nck1 LacZ (KO) Fw	ctgattgaagcagaagcctgcgatg
Nck1 LacZ (KO) Rv	tattggcttcatccaccacatacagg
Nck2 WT Fw	ctacactgcccagcaggaccagg
Nck2 WT Rv	cacatacagatacacacacgctgaag
Nck2 KO Fw	ccaatggcaaaggtgatcatgacgg
Nck2 KO Rv	cgccttctatcgccttcttgacgag

Supplemental Table 2: Primer information for quantitative PCR

Gene Name	Gene Accession	Primers (FW & RV)	Size	Secondary structure	Source
Akt1	NM_001165894	atgaacgacgtagccattgtg	116	OK	Primer bank 6753034a1
		ttgtagccaataaaggtgccat			
Esr1	NM_001302533.1	gcacattccttccttccgtct	88	OK	Jones Lab
		tgctggttcaagagcgtc			
Gapdh	NM_001289726.1	aggtcggtgtgaacggatttg	95	OK	Jones Lab
		ggggtcgttgatggcaaca			
Hprt	NM_013556.2	catggactgattatggacaggactg	126	OK	Jones Lab
		atccagcaggtcagcaaagaact			
Igf1	NM_001111274	ctggtggatgctcttcaggttcg	180	OK	Zhang et al. 2007) [43] (see bottom)
		tgcttttgtaggcttcagtggg			
Itgb1	NM_010578	ggtgtcgtgtttgtgaatgc	268	OK	Jones Lab
		tcctgtgcacacgtgtctt			
Nck1	NM_010878.3	gcggtcctcaggtgactgg	199	OK	Jones Lab
		attcatggaatttcgaactcgccacc			
Nck2	NM_010879.3	ctacactgcccagcaggaccagg	148	OK	Jones Lab
		cacatacagatacacacacgctgaag			
Pgr	NM_008829.2	ctccgggaccgaacagagt	122	OK	Jones Lab
		acaacaaccctttggtagcag			
Vim	NM_011701.4	cggctgcgagagaaattgc	124	OK	Primer bank 31982755a1
		cttgccactcagggaagga			

43. Zhang J, Moats-Staats BM, Ye P, D'Ercole AJ. Expression of insulin-like growth factor system genes during the early postnatal neurogenesis in the mouse hippocampus. J Neurosci Res. 2007;85:1618–27.
